# Internet Risk Perception: Development and Validation of a Scale for Adults

**DOI:** 10.3390/ejihpe12110111

**Published:** 2022-11-01

**Authors:** Norma Torres-Hernández, Inmaculada García-Martínez, María-Jesús Gallego-Arrufat

**Affiliations:** Department of Didactics and School Organization, Faculty of Education, University of Granada, Campus de Cartuja s/n, 18071 Granada, Spain

**Keywords:** adults, internal consistency, Internet Risk Perception Scale, online perceived risk, psychometrics

## Abstract

Despite the importance of Internet risk perception, no instrument currently exists that measures this awareness in the Spanish population. The goal of this study was to provide information on studies of the validity and reliability of the Internet Risk Perception (IRP) Scale for adult Spanish citizens. We began with a literature review and validation using a mixed panel with 20 participants. We analyzed the degree to which the subjects agreed or disagreed with the criteria evaluated, including contributions for improving the instrument, and performed a pilot test with 517 adults aged 18 to 77. Construct reliability and validity were analyzed using various statistical analyses. The results from the confirmatory factor analysis showed a sufficient accuracy of the data with parameters that indicated an excellent fit for all items. The Spanish version of the scale for adults is a reliable and valid instrument for use in studies that investigate Internet risk perception in people over 18 years of age.

## 1. Introduction

The rapid integration of information and communication technologies in all aspects of everyday life and the intensive penetration of these technologies into all corners of the world make it almost impossible to live without using electronic devices [1]. The availability, speed, and ease with which we use Internet services have contributed to the evolution of society to promote its digital dimension. At the European level, data from a report on the International Telecommunication Union (ITU) in Europe [2] showed that over 98% of the population has 3G cellphone coverage. The percentage of persons who used Internet at the end of 2019 was 82.5%, and persons aged 15–24 were the group that used Internet the most (96.2%)—well above the world average of 69%.

In the Spanish context, this trend was also confirmed by a survey of the equipment and use of ICTs in Spanish households [3], which showed that 93.9% of the population, 16–74 years of age, had used the Internet in the last three months of 2021. It was also estimated that, since the COVID-19 pandemic, 83% of the Spanish population accesses the Internet every day, and 81% connects several times a day [4].

As Byrne and Burton [5] indicated, Internet access and use are important indicators of progress toward the Sustainable Development Goals (SDGs), proposed to achieve a general well-being of the population [2,6]. At the same time, we see a growing trend in exposure through which the population becomes involved in problems online, especially among young people. As young people know technology better, however, they also develop their own strategies to protect their security and privacy [7]. We thus observe that overexposure and its risks are related to other factors, such as the digital gap, differences in digital literacy, and inequality of access—factors that may even lead to specific health-related problems [8,9].

Users’ online experiences show that the Internet provides opportunities to learn digital citizenship. Some studies, in contrast, have shown the emergence of risks in content and/or context associated with Internet use. These risks include technology addictions, cyberbullying, extorsion, and the location of inappropriate sexual content [1,10,11,12,13]. Problematic behaviors also appear both on- and offline, such as psychological and emotional difficulties, alcohol consumption, and substance abuse [13,14]. The latter two have been studied widely in relation to problematic Internet use and its consequences.

The definition of online risk, closely related to the term “harm”, refers to any situation that involves a likelihood of a violation of a user’s life when surfing the net. It focuses on the likelihood of something happening and the magnitude of its consequences [15]. It is socially constructed through information processes, social environments, and the responses that each individual gives to certain situations in their relationship with them; according to Weber [16], it could be said that familiarity with some domain or situation decreases their perception of it. This occurs in such a way that a perceived risk will be higher when a user makes a decision without certainty and will decrease, according to Purkait, Kumar, and Stuart [17], to the extent that there is more information or depending on some sociodemographic factors such as age, education, and gender.

In relation to the perception of online risks among Internet users, there is little understanding of what actually constitutes a risky situation according to Wood and Wheatcroft [18], and it is common for many to have experienced some form of harm or negative experiences such as online aggression, search redirection to inappropriate content, data and identity theft, sextortion, and grooming [19]. Yet, when asked about it, they are often conflicted in their statements.

Daily use of the Internet makes users vulnerable because it is through the Internet that they receive information and are therefore more exposed to different types of risks related to communication, e-commerce, data exposure, personal data, entertainment, and content. The Internet is the main source of information and has a high potential to affect the quality of life of Internet users [20].

A psychological approach considers some variables such as addiction, obsession, neglect, aggression, lack of control, and emotions. Risk perception can vary according to personality and age, but there are also other factors such as a lack of education, knowledge, or understanding, which can help in understanding why people take more risks and if they are more vulnerable when using the Internet.

In relation to age, an adult can identify different levels of risk depending on the situation at hand and the area that is affected, including financial, recreational, ethical, social, and health [21], and the risks could be the subject of concern and alarm, while for young people they could be more imperceptible [22].

Studies of the risks and problems associated with Internet use and technology from a socio-educational perspective report that Internet users face such situations independently of any distinguishing socioeconomic, cultural, or generational characteristics [23,24,25]. This finding shows that the entire population is at risk when surfing digital environments. The key thus lies in fostering practices that increase in individuals’ and groups’ awareness of and ability to identify the situations that lead to risks and problems [26].

Our literature review of Internet risk perception contained little evidence of the properties of questionnaires and scales used to measure these variables. Existing studies are instead associated more directly with problematic Internet use, including some items or dimensions related to Internet risks. Table 1 summarizes the main instruments found that examine digital risks in some way. 

The analysis of the studies showed a direct relationship between advances in technology and the risks of digital society due to technology use, as well as a lack of instruments to provide an accurate diagnosis of the situation. In response to this social concern, various initiatives and programs have emerged and been developed to educate and prevent risk in the general population [34,35]. One recent initiative in Spain is the project, “Development and Optimization of Intergenerational Educational Activities to Promote Responsible Internet Use” (“Desarrollo y optimización de acciones educativas intergeneracionales para la promoción del uso responsable de Internet” (EduACD)), performed during 2020–2022 to foster training actions for citizens. The instrument whose psychometric properties are presented here was developed within the framework of EduACD.

The goals of the present study were: (1) to analyze the content validity of the EPRI questionnaire in the adult population through the judgment of experts using the Delphi technique; (2) to establish the degree of understanding of the EPRI questionnaire by administering it to a sample of adults; (3) to perform analyses of the scale; and (4) to confirm the instrument’s three-dimensional character using confirmatory factor analysis (CFA).

## 2. Materials and Methods

Experts’ judgment is commonly used as an instrument in the process of evaluating and validating questionnaires and scales in education research. It is also one of the fundamental pillars of the Delphi methodology [36]. The expert judgment method is used to design tools in research studies [37,38]. After analyzing the limitations of the revised questionnaires and instruments, we decided to develop the IRP scale, that it is available in the Appendix A We established the prior requirements related to the theoretical grounding, extent, accessibility of application, clear phrasing, and adaptation of the scale to the inherent characteristics of the sample, brevity of the items, and avoidance of use of negations [39].

### 2.1. General Procedure for Developing the IRP Scale

Throughout the scale construction process, the researchers followed the conditions for an evaluation instrument proposed by Cronbach [40] and Urbina [41]. Content was determined by a scientific literature review and expert opinion [42]. We also followed the established recommendations and constructed the items using closed questions with five response options.

We began with a brainstorm by the group of researchers in the EduACD project to prepare the initial set of items to compose the IRP scale, using as a foundation, the scale developed by Byrne et al. [27] and the prior literature review. We drafted a first, experimental version adapted to the target population, reducing its complexity for the comprehensive understanding of the questionnaire. The 50 items composing the questionnaire had to be answered by the following options: “not risky at all”, “slightly risky”, “quite risky”, “very risky”, and “too risky”. The scale was then judged by experts, who were given the task of discussing and reformulating each question based on the criteria of clarity, importance, and pertinence.

### 2.2. Content Validity of the Instrument

To reach optimal levels of content validity, we followed the methodology for content validation proposed by Pozo et al. [43], with procedures for judgment by experts and apparent validation, respectively.

Using expert judgment as an instrument in the process of evaluating and validating scales and questionnaires is common practice when designing tools and instruments from numerous educational studies [44,45]. It is also one of the fundamental pillars of the Delphi methodology [36].

In this case, we invited a group of 10 experts with sufficient knowledge of the validation technique. All held PhDs—a high communicative competence [46]—in areas linked to the research problem and possessed extensive professional experience [38]. To ensure parity, 44% were men and 56% were women, with an average of 20 years’ experience in education research and instruction in higher education. The group was asked to evaluate the initial information, as well as the items proposed, and a subsequent general evaluation of each item was conducted based on the proposed criteria. Ten persons participated in the second procedure as non-expert citizens. They were asked to collaborate in evaluating the same criteria as the judges. This procedure, known as apparent validity, is used when content is being evaluated by a population group with characteristics similar to possible users of the instrument.

The phase analyzing the qualitative data used content analysis and descriptive statistics. Both the experts’ judgment and the case of apparent validity required agreement on at least 70% of the items for the three criteria evaluated. This procedure verified that the judges and non-expert validators considered most of the items clear, pertinent, and important. An analysis of the value of the experts’ agreement on the criterion of importance enabled us to conclude significant agreement among the evaluating judges (0.539), with a resulting intensity of Kendall’s W = 0.088. The same analysis of the non-experts showed significant agreement among the non-experts for all items, with a resulting intensity of Kendall’s W = 0.178.

### 2.3. Pilot Application

Considering the Spanish population of over 18 years of age, we calculated the sample with a confidence level of 95%. The recruitment process of the sample was performed through different channels (university and social networks). The only inclusion criterion used was that they should be of legal age. Moreover, the fact that the instrument was aimed at the general population made it difficult to elaborate a more detailed profile or grouping in terms of the similarity of the population. The scale was administered to 517 subjects aged 18–77 (M = 44–78; DT = 13–837). Of these, 194 were men (37.5%) and 323 were women (62.5%).

After the phase of content validation and the pilot test of the questions proposed, we examined a series of statistical indicators, such as the discrimination index and descriptive statistics for each item. To provide sufficient accuracy of the data obtained, we also considered it pertinent to conduct studies of reliability and validity. The validity study fulfilled the psychometric requirements, with a satisfactory Cronbach reliability coefficient and subsequent CFA [40].

### 2.4. Data Analysis

Treatment and analysis of the quantitative data included a descriptive analysis, as well as an evaluation of internal consistency using the statistical package SPSS 25.0. Next, the program, FACTOR Analysis 9.3.1, was used for exploratory factor analysis (EFA) and M-PLUS 7, for CFA.

## 3. Results

To obtain results related to the instrument’s content validity, we followed various qualitative methods to obtain scientific evidence on the efficacy of the concept, culture, and language of the IPR scale.

The qualitative data were complemented with quantitative ratings the validators attributed to each item. Such integration of comments formalized by both groups also constituted a dichotomy of independent positions to guarantee the scale’s pertinence and appropriateness.

The 50 items composing the questionnaire were revised to adapt them to the characteristics of the study population, as reflected in the validators’ scores for each item.

SPSS 25.0 and FACTOR Analysis 9.3.1 were used for EFA. First, data analysis was performed by calculating the descriptive values of each item [46]. The analytical process showed that no item had to be eliminated because it did not obtain values above 2 in the tests of dispersion, asymmetry, and kurtosis (Table 2).

Table 3 presents the rotated loading matrix, created with the statistical program, FACTOR Analysis [47]. Bartlett’s test statistic (5739.2 (df = 1225; *p* = 0.00010)) and the results of the Kaiser–Meyer–Olkin (KMO) test (=0.945), used to test whether the sample came from populations with the same variance and whether a good sample fit was present, indicated a good fit of the data analyzed in the factor analysis [48]. The three factors extracted explained 68.1% of the variance, the comparative fit index (CFI) was 0.996, the goodness-of-fit index (GFI) was 0.984, the adjusted goodness of fit index (AGFI) also took the value 0.982, and the root mean square residual (SRMR) took a value of 0.042. These parameters indicated an excellent fit of all items. Finally, to analyze reliability, we calculated the value of Cronbach’s alpha, obtaining 0.937 for the scale in general and values above 0.400 for all factors, except 22 and 33, which took values of 0.364 and 0.390, respectively.

Table 4 shows Pearson’s correlation between the three IRP scale dimensions. The dimension, “Privacy and data protection” showed a strong correlation with “Communication risks with people and entities” (r = 0.892) and with “Behavioral risks” (r = 0.737). Likewise, “Safe internet use” and “Internet risks” obtained a correlation of r = 0.858.

**Figure 1:**
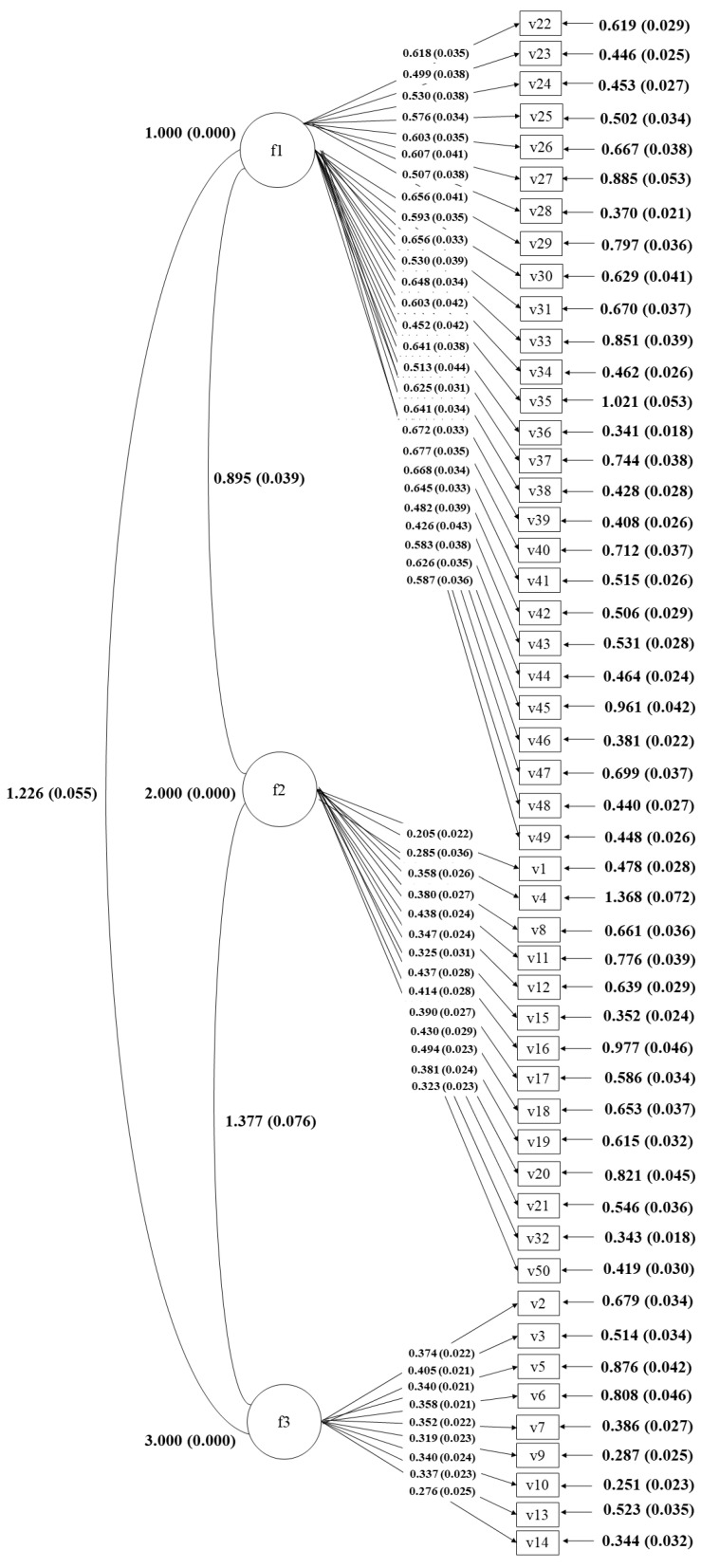
CFA of the IRP scale.

After performing the EFA and obtaining the data on reliability for each item, we obtained the instrument’s validity through CFA. To achieve this, we grouped the 50 questions into a three-dimensional structure: F1 = privacy, data protection, and digital security; F2 = relationship with people, digital content, and entities; and F3 = harmful behavior. The model results showed absolute confirmation of the model. On the other hand, by confirming the indices that infer model fit, we obtained a good fit. From examining the values of the different indices, the CFI contributed a value of 0.786, while the TLI was 0.776. The Chi-square took a value of 11,552.977 with 1225 degrees of freedom. The SRMR took a value of 0.068. Finally, the RMSEA value indicated an acceptable model fit, with a value of 0.060. Since these parameters served as a fundamental pillar in the analysis, Figure 1 confirmed that the proposed model showed a reasonable and acceptable approximation of the data, thus contributing to the solidity and verification of the three-dimensional hypotheses of the construct tackled. To conclude, we reviewed the analysis of the proposed factor model. Figure 1 presents the estimations of the factor saturations obtained for each item.

## 4. Discussion and Conclusions

Based on the data obtained, “Privacy and data protection” showed a strong correlation with “Safe internet use”. The developed instrument can therefore also be considered a useful and relevant research tool for the study of risk practices associated with the dimensions obtained in the CFA, such as personal data protection [49,50], communication risks with people and entities [51], and behavioral risks [5]. These three categories are among some that the OECD has classified as potential risks on the Internet, which are also very much in line with elements of Livingstone’s and Stoilova’s [52] “4Cs” risk proposal. Moreover, according to some studies, these dimensions are some of the most common concerns of citizens today.

This scale also provides an organizational framework on risks which can be useful to understanding the degree of knowledge that adults have about different practices on the Internet and to determining whether or not they are perceived as risks.

On the other hand, as suggested by some authors [23,24,25,26], the scale designed as part of socio-educational research considers variables that can be related to cultural or generational characteristics.

Despite the advantages it provides, due to the scarcity of previous studies related to the construction and validation of scales that study risks on the Internet, this scale can be considered an instrument for approaching the object of study with its own limitations in the social sciences. Therefore, its results cannot be generalized due to the variety of factors and variables that may influence its application and the possible answers given by the subjects investigated.

We know that a better understanding of any issue in digital society research requires new, reliable, and valid instruments which provide more accurate information to differentiate normal from problematic use, as well as to distinguish the types of risks that require psychological attention from risks that can be addressed and prevented through digital safety education. This validated scale is therefore a useful tool for defining actions in the fields of education, family, and social life [26,27,28,29,30,31,32,33,34], which can help to reduce the exposure of adults to risks that are becoming more frequent due to the increase in daily Internet use time. This usefulness is based on the need raised by Dönmez et al. [29], who pointed out that society frequently demands that policymakers assess the risks of technologies and manage them appropriately in social integration processes.

### Limitations and Future Studies

Despite the high indices of reliability and validity reported by the instrument developed, this study was not free of limitations. The first involved the heterogeneity of the samples, as the selection criterion only required that participants be older than 18, leading to too wide of an age range. Nor did we consider the participants’ profession and education level, potentially generating bias by omitting participants’ level of literacy and/or digital competence. The second limitation involved the instrument itself. We found that the absence of prior instruments to evaluate the variables helped to place too much stress on the conceptual frameworks for Internet security and risk.

Future studies must attempt to incorporate more questions attempting to characterize the participants better to reduce the potential gaps identified. We will thus proceed to administer various versions of the instrument addressed to more specific populations in terms of occupation (university students and working citizens) or age ranges (young people, adults, and older people). Related to the results of the application of the scale, another possible route would be to analyze the relationships between variables, such as age, gender, employment status, and level of studies, with the three factors resulting from the factor analysis: privacy, data protection, and digital security; the relationship between people, digital content, and entities; and harmful behaviour.

Another aspect of interest is conducting research into the relationship between risk perception and its impact on users’ quality of life. The relationship between digital competence and levels of risk perception in the three factors identified in the scale is another point to explore.

## Figures and Tables

**Table 1:** Studies of risk perception.

Author	Title	Instrument	Variables	Population
Byrne et al. [27]	From the user’s perspective: Perceptions of risk relative to benefit associated with using the Internet	Interviews, 35 items	(1) Preparation of a list of 35 Internet activities; (2) users’ perception of risk associated with each action; (3) users’ evaluation of the frequency with which they performed the action; (4) benefits that users believed they obtained from the action; and (5) the quantity of personal information users were willing to share to obtain a benefit	261 adults aged 50–64
Demetrovics et al. [28]	The three-factor model of Internet addiction: the development of the Problematic Internet Use Questionnaire	Problematic Internet Use Questionnaire (PIUQ), 30 items	Obsession, negligence, and control disorder	1.037 persons (54.1% men; average age, 23.3)
Dönmez et al. [29]	Development of a Scale to Address Perceptions of Pre-service Teachers Regarding Online Risks for Children	Questionnaire on problematic Internet use developed by Demetrovics et al. (2008), 25 items	Sexuality, online account, cyberbullying, inappropriate content, dangerous communications, and revelation of confidential information	Turkish education students; no information on age
Montiel et al. [30]	Analysis of a brief scale of Internet risk behavior in Chilean youth	Questionnaire on the online victimization of minors (i.e., the JOVQ), 13 items	Bold contact with strangers and indirect risk	Persons aged 15–19
Jelenchick et al. [31]	Screening for Adolescent Problematic Internet Use: Validation of the Problematic and Risky Internet Use Screening Scale (PRIUSS)	Problematic and Risky Internet Use Screening Scale (PRIUSS), 18 items	Social deterioration, emotional deterioration, and risky/impulsive Internet use	University students aged 18–25
Kelley and Gruber [32]	Problematic Internet Use Questionnaire	Adaptation of Demetrovics et al. (2008), 18 items	Obsession, negligence, and control disorder	278 students aged 18–37
Milková and Ambrožov [33]	Internet Use and Abuse: Connection with Internet Addiction	Learning Combination Inventory from Internet Risks Questionnaire (IRQ) (Kalibova, 2017), 28 items	The Sequential Processor The Precise ProcessorThe Technical Processor learning pattern student The Confluent Processor learning pattern student	1542 students aged 15–23

**Table 2:** Descriptive analysis of the IRP scale.

Variable	M	CI (95%)	V	S	K	Variable	M	CI (95%)	V	S	K
**V1**	2.273	(2.19–2.36)	0.570	0.665	1.166	**V26**	3.524	(3.41–3.64)	1.031	−0.121	−0.697
**V2**	3.615	(3.50–3.73)	1.111	−0.424	−0.271	**V27**	3.551	(3.43–3.68)	1.249	−0.216	−0.997
**V3**	4.182	(4.07–4.29)	1.008	−1.198	0.942	**V28**	4.453	(4.36–4.54)	0.635	−1.455	1.851
**V4**	2.497	(2.36–2.64)	1.538	0.741	−0482	**V29**	3.801	(3.68–3.93)	1.227	−0.524	−0.661
**V5**	3.021	(2.90–3.15)	1.228	0.266	−0.694	**V30**	3.509	(3.40–3.62)	0.981	−0.186	−0.495
**V6**	3.729	(3.61–3.85)	1.196	−0.364	−0810	**V31**	3.725	(3.61–3.84)	1.101	−0.310	−0.906
**V7**	4.416	(4.32–4.51)	0.757	−1549	1.268	**V32**	2.226	(2.14–2.32)	0.635	0.926	1.481
**V8**	2.638	(2.53–2.75)	0.927	0.607	−0.019	**V33**	3.406	(3.29–3.53)	1.139	−0.022	−0.949
**V9**	4.576	(4.49–4.66)	0.604	−1.077	1.482	**V34**	3.975	(3.87–4.08)	0.883	−0.566	−0.320
**V10**	4.634	(4.55–4.72)	0.599	−1.371	1.602	**V35**	3.574	(3.44–3.71)	1.386	−0.297	−0.992
**V11**	2.849	(2.73–2.97)	1.072	0.452	−0.453	**V36**	4.617	(4.53–4.70)	0.546	−1.782	1.909
**V12**	3.389	(3.27–3.50)	1.023	−0.027	−0.789	**V37**	3.627	(3.51–3.75)	1.159	−0.344	−0.630
**V13**	4.271	(4.17–4.38)	0.871	−1.164	0.681	**V38**	4.518	(4.42–4.61)	0.691	−1.922	1.612
**V14**	4.619	(4.53–4.70)	0.576	−1.241	1.074	**V39**	4.112	(4.01–4.21)	0.800	−0.693	−0.129
**V15**	2.199	(2.11–2.29)	0.597	1.129	1.990	**V40**	3.861	(3.74–3.98)	1.122	−0.483	−0.824
**V16**	3.675	(3.55–3.80)	1.202	−0.407	−0.696	**V41**	3.714	(3.60–3.82)	0.966	−0.295	−0.620
**V17**	2.816	(2.71–2.93)	0.966	0.546	−0.235	**V42**	3.727	(3.62–3.84)	0.964	−0.230	−0.731
**V18**	2.868	(2.76–2.98)	0.996	0.663	−0.279	**V43**	3.652	(3.54–3.76)	0.977	−0.185	−0.655
**V19**	2.364	(2.26–2.47)	0.920	1.024	0.891	**V44**	3.706	(3.60–3.81)	0.881	−0.270	−0.440
**V20**	2.843	(2.72–2.97)	1.192	0.555	−0.524	**V45**	2.928	(2.81–3.05)	1.192	0.366	−0.662
**V21**	2.855	(2.74–2.97)	1.033	0.437	−0.337	**V46**	4.563	(4.48–4.65)	0.575	−1.801	1.912
**V22**	3.838	(3.72–3.95)	1.006	−0.373	−0.702	**V47**	3.526	(3.41–3.64)	1.038	−0.071	−0.804
**V23**	4.441	(4.35–4.54)	0.699	−1.394	1.308	**V48**	4.062	(3.96–4.17)	0.839	−0.667	−0.130
**V24**	4.377	(4.28–4.47)	0.734	−1.307	1.153	**V49**	4.104	(4.00–4.20)	0.790	−0.653	−0.260
**V25**	4.000	(3.90–4.10)	0.832	−0.567	−0.215	**V50**	3.251	(3.16–3.34)	0.625	0.227	0.593

Note: Mean (M); confidence interval (CI); variance (V); skewness (S); kurtosis (K).

**Table 3:** Factor loading on the IRP scale.

Variables	F1	F2	F3
V 01		0.460	
V 02			0.569
V 03			0.678
V 04		0.413	
V 05			0.438
V 06			0.543
V 07			0.689
V 08		0.524	
V 09			0.726
V 10			0.718
V 11		0.492	
V 12		0.409	
V 13			0.409
V 14			0.533
V 15		0.691	
V 16		0.578	
V 17		0.546	
V 18		0.514	
V 19		0.669	
V 20		0.508	
V 21		0.596	
V 22	0.364		
V 23	0.474		
V 24	0.575		
V 25	0.550		
V 26	0.461		
V 27	0.432		
V 28	0.609		
V 29	0.490		
V 30	0.633		
V 31	0.583		
V 32		0.688	
V 33	0.390		
V 34	0.603		
V 35	0.416		
V 36	0.612		
V 37	0.421		
V 38	0.608		
V 39	0.651		
V 40	0.634		
V 41	0.708		
V 42	0.747		
V 43	0.710		
V 44	0.800		
V 45	0.405		
V 46	0.702		
V 47	0.622		
V 48	0.734		
V 49	0.686		
V 50		0.485	

**Table 4:** Pearson’s correlation of the dimensions of the IRP scale.

	Privacy and Data Protection	Communication Risks with People and Entities	Behavioral Risks
Privacy and data protection	1	0.892 **	0.737 **
Communication risks with people and entities		1	0.858 **
Behavioral risks			1

** Correlations are significant at level 0.01 (bilateral).

## Data Availability

The datasets generated during and/or analyzed during the current study are available from the corresponding authors upon reasonable request.

## Appendix A. English Version of the Validated Instrument

How risky do you perceive the Internet to be?

The response options for items 1 to 50 are as follows on the Likert scale:

- Not at all risky
- Slightly risky
- Fairly risky
- Very risky
- Too risky

1. Accessing personal or professional websites.
2. Accessing matchmaking sites.
3. Easily accessing sexual or pornographic websites.
4. Arranging a date with people you know.
5. Gambling on the Internet.
6. Keeping in contact with unknown people through social networks (Facebook, Instagram, or others).
7. Posting personal information on the Internet that could be used to harm me.
8. Accessing interactive online entertainment (videos, chats, series, films, etc.).
9. Sharing photographs or videos of minors.
10. Sharing sexually provocative images.
11. Downloading images or photographs.
12. Sharing photos on the Internet.
13. Using information, photographs, or videos without permission.
14. Giving my bank or credit card details on gambling or gaming sites.
15. Shopping on websites such as Amazon.
16. Searching for consumer or user reviews of products and being asked for personal details.
17. Accepting cookies to continue browsing the Internet.
18. Receiving advertising via e-mail or social networks.
19. Making transactions on the bank’s website.
20. Making online transfers through entities such as Western Union.
21. Accepting privacy policies when registering on social networks or apps.
22. Sharing personal information or data (name, age, telephone number, location, etc.).
23. Sharing login passwords with other people.
24. Misplacing a pen (pen drive) containing personal information.
25. Not knowing what personal data are managed and shared by companies.
26. Not changing my passwords from time to time.
27. Using public Wi-Fi networks.
28. Seeing personal information published without my consent.
29. Having my webcam uncovered.
30. Accessing health, food, and consumer information without being sure of its veracity.
31. Not logging out when I finish using accounts or profiles.
32. Communicating via WhatsApp.
33. Sharing and disseminating private messages to contacts or groups on social networks.
34. Making private information public on profiles created on the Internet.
35. Creating a fake profile on a social network.
36. Inducing others to perform embarrassing or indecent acts in front of a webcam and then publishing it.
37. Sharing personal information with my contacts instead of face-to-face.
38. Using social networking sites to spread rumors, insult, or threaten others.
39. Not knowing how to manage my passwords securely and appropriately.
40. Opening junk e-mail (spam).
41. Failing to acknowledge authorship of something copied.
42. Downloading unlicensed tools, programs, or applications.
43. Downloading music or games without verifying their origin or authorship.
44. Not knowing what to do when pop-up windows or advertisements appear on the Internet.
45. Exchanging files between work, school, or home computers.
46. Downloading apps, programs, or materials that have viruses.
47. Giving permission to make changes to the computer when using a program.
48. Clicking on links without knowing if they are safe.
49. Not knowing how to recover information in the event of theft or loss.
50. In general, I perceive the Internet to be (not at all–a little–quite a lot–very–extremely) risky.
